# Enhanced charge carrier transport properties in colloidal quantum dot solar cells *via* organic and inorganic hybrid surface passivation†
†Electronic supplementary information (ESI) available. See DOI: 10.1039/c6ta06835a



**DOI:** 10.1039/c6ta06835a

**Published:** 2016-10-07

**Authors:** John Hong, Bo Hou, Jongchul Lim, Sangyeon Pak, Byung-Sung Kim, Yuljae Cho, Juwon Lee, Young-Woo Lee, Paul Giraud, Sanghyo Lee, Jong Bae Park, Stephen M. Morris, Henry J. Snaith, Jung Inn Sohn, SeungNam Cha, Jong Min Kim

**Affiliations:** a Department of Engineering Science , University of Oxford , Oxford OX1 3PJ , UK . Email: junginn.sohn@eng.ox.ac.uk ; Email: seungnam.cha@eng.ox.ac.uk; b Department of Physics , Clarendon Laboratory , University of Oxford , Oxford OX1 3PU , UK; c Jeonju Centre , Korea Basic Science Institute , Jeonju , Jeollabuk-do 561-180 , Republic of Korea; d Department of Engineering , University of Cambridge , Cambridge CB3 0FA , UK

## Abstract

Colloidal quantum dots (CQDs) are extremely promising as photovoltaic materials. In particular, the tunability of their electronic band gap and cost effective synthetic procedures allow for the versatile fabrication of solar energy harvesting cells, resulting in optimal device performance. However, one of the main challenges in developing high performance quantum dot solar cells (QDSCs) is the improvement of the photo-generated charge transport and collection, which is mainly hindered by imperfect surface functionalization, such as the presence of surface electronic trap sites and the initial bulky surface ligands. Therefore, for these reasons, finding effective methods to efficiently decorate the surface of the as-prepared CQDs with new short molecular length chemical structures so as to enhance the performance of QDSCs is highly desirable. Here, we suggest employing hybrid halide ions along with the shortest heterocyclic molecule as a robust passivation structure to eliminate surface trap sites while decreasing the charge trapping dynamics and increasing the charge extraction efficiency in CQD active layers. This hybrid ligand treatment shows a better coordination with Pb atoms within the crystal, resulting in low trap sites and a near perfect removal of the pristine initial bulky ligands, thereby achieving better conductivity and film structure. Compared to halide ion-only treated cells, solar cells fabricated through this hybrid passivation method show an increase in the power conversion efficiency from 5.3% for the halide ion-treated cells to 6.8% for the hybrid-treated solar cells.

## Abstract 2

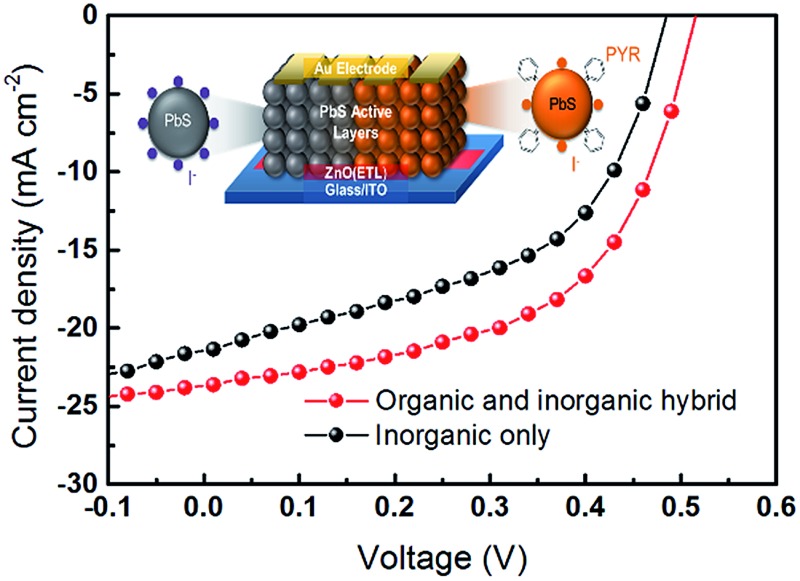
We report a PbS QD hybrid passivation structure to eliminate trap sites while increasing charge extraction in QD solar cells.

## Introduction

The remarkable features of colloidal quantum dots (CQDs) have led to their deployment in a range of potential technological applications.^1–4^ Specifically, the tunability of the band gap (ε_gap_) along with their compatibility with solution processing makes them one of the most promising materials for solar energy harvesting.^5–7^ Moreover, lead-based quantum dots such as lead sulfide (PbS) and lead selenide (PbSe) have been shown to be, potentially, the most attractive CQDs for solar cell devices, possessing an optimal ε_gap_ of ∼1.3 eV thus resulting in absorption at infrared wavelengths.^8^ Recently, the highest power conversion efficiencies (PCEs) of quantum dot solar cells (QDSCs) that have been reported have exceeded 10%, and research on QDSCs has continued to demonstrate considerable efficiency improvements in recent years compared with other types of PVs.^9,10^ Continued improvements in QDSCs mainly rely on the judicious control of the surface passivation by replacing the initial, long-chain aliphatic ligands on the CQDs with smaller molecules.^11–13^ This ligand exchange process is considered to be a critical step in the fabrication of CQD films and it has also been shown to have a significant impact on the resulting electrical properties.^14,15^ For instance, insufficient passivated CQD films will still consist of the initial bulky ligands thereby resulting in low charge mobility and conductivity; it can also make them less stable due to exposure to air. Furthermore, failure to accomplish the surface passivation will cause a drastic decrease in the photoluminescence quantum yield as well as unwanted fast carrier recombination, which is believed to result from the generation of surface trap sites.^16^ Therefore, in QDSCs, enhancing carrier transport properties and minimizing the degree of surface trap sites are the major issues that have to be resolved, which in turn relies on a better surface passivation process.

To date, the prevalent ligands used in QDSCs are acknowledged to be atomic halides such as Cl^–^, Br^–^ and I^–^ because of a good metal Pb and halide ion coordination. Strong surface passivation with halide ions and superior charge carrier diffusion within the CQD films can be achieved, which ultimately leads to significant enhancements in the PV performance. Generally, most of these halide treatments are prepared through a solid-state exchange process.^17,18^ Nevertheless, it has been noted that the initial capping ligands on the CQDs cannot be fully replaced through this solid-state exchange process. Recently, Balazs *et al.* ^19^ reported that the conventional ligand exchange process using tetrabutylammonium iodide (TBAI) dissolved in methanol cannot lead to a near-complete removal of the initial ligands (oleic acids) in a short time scale. This was found to be responsible for the low carrier mobility and disordered packing densities in the CQD films. Moreover, the work by Bawendi's group has demonstrated that the discrepancy between the open-circuit voltage (*V*_oc_) and the band gap of the CQDs resulted from the existence of sub-ε_gap_ states, which were originally generated from the charged Pb atoms that remained after the halide ligand exchange process.^20,21^ Hence, there is still considerable scope for further improvement so as to attain a high level of performance in terms of device parameters such as the short circuit current density (*J*_sc_), open circuit voltage (*V*_oc_) and overall PCEs. To this end, improved passivation strategies that efficiently remove the initial ligands and reduce the surface trap sites are still highly sought after. Here, we propose the use of a hybrid halide ion (*i.e.* TBAI) with pyridine to passivate the CQD surface with the intention of improving the performance of QDSCs. In this study, we have chosen to use pyridine as one of the ligands as it is known to be one of the smallest soft base molecules with an amine anchor group and it has already been successfully applied in various CQD optoelectronic devices for the purposes of surface passivation.^22,23^ Its short molecular length can increase the carrier mobility following CQD passivation whereas its alkalinity facilitates a near-complete removal of the pristine surfactant (*e.g.* Oleic acids) during the fabrication of CQD films. In addition, pyridine can create favorable binding with Pb metal atoms, and eventually decrease any surface defects.^24^ By combining two short ligands (TBAI and pyridine in this case) we are able to reduce the formation of sub-ε_gap_ states and the trap sites through pyridine passivation as well as maintaining a high exciton diffusion channel as a result of the halide ion functionalization. Encouragingly, we successfully show reduced non-radiative recombination and lowered trap sites using this hybrid passivation process. We also demonstrate that an enhanced PCE can be readily achieved in our hybrid planar single junction solar cells in contrast to a TBAI-only reference cell. The improved PCE is found to result from the enhancement of the *J*_sc_, *V*_oc_, fill factor and CQD solid film parameters.

## Results and discussion

**Fig. 1:**
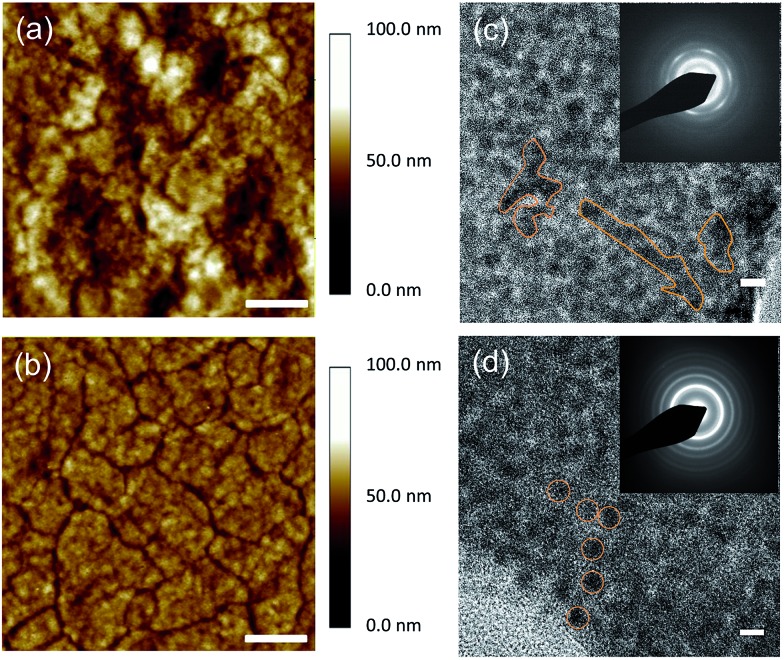
AFM images of the PbS quantum dot films with (a) TBAI and (b) Hybrid (TBAI + Pyr) passivation, scale bars are 2 μm. The average root-mean-square (RMS) roughness of the HB and TBAI CQD films is 4.465 nm and 9.132 nm, respectively. HRTEM images of the PbS quantum dot films with (c) TBAI and (d) hybrid (HB, TBAI + Pyr) passivation, scale bars are equal to 5 nm. Inset: SAED patterns of the corresponding PbS quantum dot films.

In order to investigate the influence of the different surface passivation treatments on the PbS CQD films between TBAI only and hybrid (HB, TBAI + pyridine) films, atomic force microscopy (AFM) and transmission electron microscopy (TEM) analyses of the PbS CQD films were carried out. Fig. 1a and b show the AFM topography images of TBAI-treated and HB-treated CQD films, respectively. It can be clearly observed that the two PbS CQD films show very different film morphologies. The TBAI-treated PbS CQD films show a non-uniform surface consisting of significant cracks and roughness in contrast to the relatively flat and uniform surface with fewer cracks and mild roughness from the HB-treated PbS CQD films. For completeness, images of the 3D CQD film topology obtained using AFM are also provided (Fig. S1†).

Insufficient removal of the ligands on the quantum dots can result in an irregular spacing as well as aggregation of the quantum dots, which can lead to extensive cracking of the CQD films.^25^ Therefore, in this case, the AFM images indicate that the CQD films after the HB surface passivation exhibit a more uniform spacing of the quantum dots and less aggregation leading to a more even distribution. Fig. S7† presents AFM images for different locations on the HB and TBAI CQD films, and Table S1† provides the average RMS values of the films. These distinct features of the CQD surface can be further evaluated after zooming in to the detailed crystal texture based on TEM images in order to better understand the passivation effects. As illustrated in Fig. 1c and d, the TBAI-treated PbS films show highly fused and aggregated features as highlighted in the HRTEM image, which is consistent with selected area electron diffraction (SAED) patterns shown in the inset where the concentric ring patterns are discontinuous. Large crystal grain domains, ranging from ∼10 nm to ∼50 nm, can also be seen in the TBAI CQD films. Indeed, the SAED analysis provides clear evidence that the TBAI treated film generated a large amount of fused QD aggregation, identified by the ring type patterns, which will inevitably lose their quantum confinement due to the large crystal domains.^26^ It is not expected that these large crystal grains would exhibit any PL emission that would resemble that of the pristine QDs.^27^

**Fig. 2:**
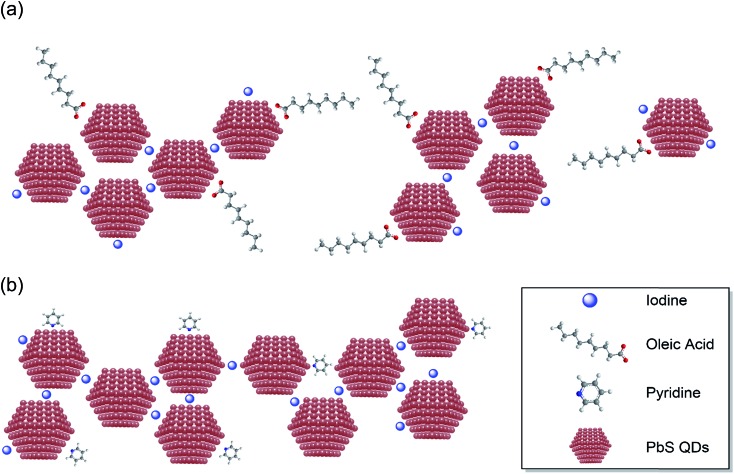
Illustrations of the QD assembly patterns for the (a) TBAI and (b) hybrid treated PbS film functionalization.

On the other hand, the HB treatment effectively decreases the CQD inter-dot distance while maintaining a good dispersity across the CQD films. As highlighted in the HRTEM images, individual PbS CQDs can be resolved in the HB solid films, which exhibit a high packing density and distinguishable crystal boundaries. Moreover, the continuous concentric ring patterns in the SAED images (shown in the inset of Fig. 1d) also indicate that the HB CQD films consist of even and small-sized film grains. This uniform topography and small grain boundaries are strongly attributed to the status of the CQD surface based on the improved passivation with pyridine. As illustrated in Fig. 2a and b, the iodine ion passivation dramatically reduces the CQD inter-dot distance although, unfortunately, it also induces a large amount of aggregation and cracking, which might impair the quantum confinement properties. However, due to the extra pyridine intercalation on the PbS surface, the HB passivation efficiently controls the CQD films allowing for a decrease in the particle separation, which substantially preserves the quantum confinement effects. We postulate that this noticeable difference in the formation of the CQD films may have a big influence on the electrical and photovoltaic properties, which will be discussed in the following sections.

**Fig. 3:**
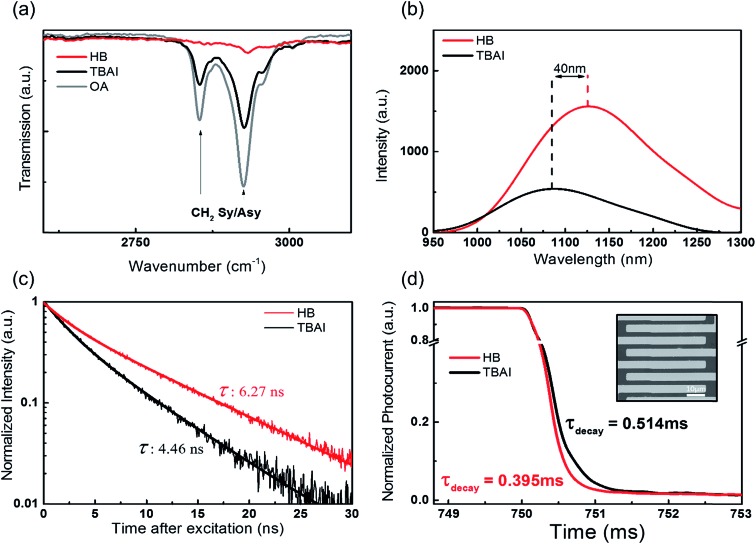
(a) FT-IR spectra of the pristine CQD film (oleic acids), TBAI and HB ligand exchanged films. (b) Stationary photoluminescence of the HB and TBAI passivated PbS CQD films with an identical thickness. (c) Time-resolved photoluminescence of HB and TBAI passivated PbS CQD films. (d) Photoresponse speeds of the PbS films with HB and TBAI passivation at a bias of 10 V and an incident power density of 4.5 mW cm^–2^. Photocurrent and decay time (*τ*) are measured under illumination of a 450 nm laser. The channel length is 5 μm with an Au electrode deposited on the 300 nm SiO_2_/Si substrate. The inset image shows the actual device.

In order to understand the surface chemical structure, the HB and TBAI passivated PbS CQD films were investigated by studying the FT-IR spectra, an example of which is shown in Fig. 3a. The as-prepared PbS CQDs with oleic acid show two dominant peaks in the FT-IR spectra, which correspond to the symmetric stretching vibration (2854 cm^–1^) and asymmetric stretching vibration (2924 cm^–1^) of –CH_2_ of the oleic acids.^28^ These two peaks are the main signatures that indicate that oleic acid remains on the CQD surface. Therefore, the absence of those two peaks implies the complete removal of the oleic acid from the CQD surface. It can be clearly observed that the –CH_2_ stretching vibrations almost completely disappear after the HB passivation, although they still can be seen in the TBAI treated films. This indicates that HB treatment is a superior process in terms of removing the oleic acid, enabling better electron charge transport in the films. Moreover, the full spectral range of the FT-IR data indicates an interaction between the pyridine and quantum dot films (Fig. S5†). To further probe the fundamental impact of the hybrid coating of the CQD films with pyridine, we studied both the steady-state and time-resolved photoluminescence (TR-PL) properties. Before the spectroscopy measurements, all the samples were prepared with the same CQDs with almost the same thickness of the CQD layers, which was confirmed by AFM measurements (Fig. S2†).

A comparison of the stationary PL and the TR-PL between the two differently treated CQD films helps us to understand the degree of surface trap sites and the difference in the trap-mediated recombination. The HB passivation can lead to a reduction in the deep trap sites, which are responsible for non-radiative recombination. In Fig. 3b, for the same thickness conditions, the stationary PL intensity of the HB treated films is higher than that of the TBAI-only treated films, which indicates an increase in the radiative recombination and a reduction of the surface trap sites. Moreover, there is a redshift in the exciton peak of the PL spectra between the HB and TBAI treated films, which suggests the enhancement of quantum coupling due to a reduction in the inter-particle spacing.^29–31^

The HB-treated CQD films display a much longer PL decay in the TR-PL measurement as shown in Fig. 3c. A prolonged exciton lifetime is an indicator that the faster, non-radiative, recombination channels have been suppressed and there is little influence from deep trap sites. The increased PL intensity and PL lifetime strongly indicate that the HB treatment results in a significant reduction of the surface trap sites and the corresponding non-radiative recombination within the CQD films. Moreover, in Fig. S6,† a red shift is observed in the absorption edge for both the TBAI and HB treatments in contrast to the colloidal solution. For the same thickness conditions, the first exciton peak intensity of the HB films is higher than that of the TBAI films, which further supports our claim that the pyridine and TBAI hybrid ligand treatment provides a better passivation compared to the TBAI-only treated films.

Measuring the rise and decay time using a photodetector allows us to compare the photovoltaic carrier transfer in the CQD films.^32^ The inset of Fig. 3d shows the simple photodetector structure upon light illumination. For the simple photodetector measurement, at a fixed bias of 10 V, a reproducible photocurrent was obtained by switching the light source on-off over a series of repeated cycles on the PbS CQD films. The decay time (*τ*) of the photoresponse obtained from the HB passivation devices was approximately 0.395 ms, which is faster than the decay time of 0.514 ms recorded for the TBAI passivation as shown in Fig. 3d. This fast decay is attributed to a strong surface interaction with the pyridine, which reduces the surface trap sites and decreases the time required to respond to the exposure from the light source. As a result of the HB treatment, we found that the PbS CQD films exhibit improved surface passivation such as the complete removal of the oleic acid, a decrease in the non-radiative decay and a reduction of the surface trap sites. Therefore, we now present a solution-processed QDSC to demonstrate these benefits in a practical photovoltaic device.

**Fig. 4:**
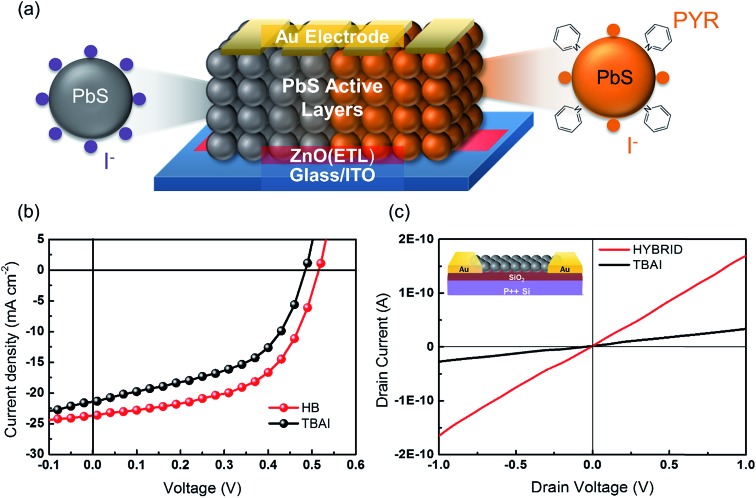
(a) Illustration of PbS QDSCS by using a layer-by-layer deposition process. (b) Current density-voltage (*I*–*V*) characteristics of the HB treated PbS film (red) and the TBAI treated PbS film (black) devices. The power conversion efficiency for the HB device is 6.8%, and that for the TBAI only device is 5.3%. (c) Conductance *I*–*V* curves of the HB and TBAI treated films that are deposited onto an Au-patterned SiO_2_ device. Inset: illustration of the SiO_2_ devices.

In order to investigate the charge carrier properties of the HB passivation in a practical device, we present solution-processed single junction QDSC devices with a ZnO electron transfer layer. Fig. 4a shows an illustration of the operating principle of the fabricated QDSCs. Typically, a ZnO nanoparticle film is used to act as an electron-accepting layer, which was spin-coated onto the indium tin oxide/glass (ITO/glass) substrate. Subsequently, CQD films and the corresponding ligand exchange treatment with the TBAI and HB were then deposited onto the ZnO films. In this case, the CQD films act as the layers responsible for generating the charge. The device fabrication was completed using 100 nm-thick Au cathodes. Fig. 4b shows the *I*–*V* characteristics under AM1.5G conditions for the champion HB and the reference TBAI treatment solar cells. Encouragingly, the total power efficiency is much improved for the HB treatment. A detailed comparison of the device parameters is provided in Table 1, where *J*_sc_ is the short circuit current, *V*_oc_ is the open circuit voltage, *R*_sh_ is the shunt resistance, FF is the fill factor and PCE is the power conversion efficiency. It can be determined that the device consisting of the 10 TBAI CQD layers shows an open circuit voltage (*V*_oc_) of 0.485 V, a short circuit current (*J*_sc_) of 21.36 mA cm^–2^, a fill factor (FF) of 0.51 and a power conversion efficiency (PCE) of 5.3%. However, by adding the extra pyridine treatment step on the PbS–TBAI active layers it appears to noticeably increase *V*_oc_, *J*_sc_ and FF resulting in an overall increase in the power conversion efficiency of 6.8%. Moreover, in Fig. S4,† *J*_sc_ determined from the external quantum efficiency (EQE) measurements also shows the same trend in terms of an enhancement of *J*_sc_ for the HB films compared with the TBAI CQD films.

**Table 1:** Performance parameters for the QDSCs under AM 1.5 spectral illumination (100 mW cm^–2^). Average values for each device with standard deviations were collected from 10 devices, and the device area of the solar cells is 0.03 cm^2^. The values in parenthesis are from the champion PCE cell

	*J*_sc_ (mA cm^–2^)	*V*_oc_ (V)	*R*_sh_	FF	PCE (%)
HB	23.64 ± 0.63 (23.55)	0.52 ± 0.01 (0.52)	127.15 ± 5.48 (132.31)	0.54 ± 0.02 (0.55)	6.59 ± 0.13 (6.76)
TBAI	20.60 ± 0.98 (21.36)	0.50 ± 0.01 (0.49)	59.73 ± 4.68 (64.12)	0.50 ± 0.02 (0.51)	5.15 ± 0.13 (5.28)

The improvement that is recorded here is consistent with the near-complete removal of the oleic acid and also the reduced surface trap sites on the CQD films. After applying pyridine onto the CQD surface, the remaining oleic acid ligands are effectively removed, resulting in a well-ordered CQD film with better surface alignment properties. Fig. 4c shows the *I*–*V* curves for an electrical conductance measurement on the TBAI and HB CQD films. The *I*–*V* curve for HB passivation is steeper than that recorded for the TBAI passivation process, which indicates that an electrically favorable environment is formed. Furthermore, these findings are consistent with other reports that have shown that hybrid passivation methods can have a significant effect on the performance of a solar cell. Due to the decrease in the inter-dot spacing, there is then an increase in the probability that the wavefunctions of the charge carriers overlap.^29,33^ Therefore, the pyridine treatment appears to have a great influence on the improved electron transport dynamics, enabling a greater overlap of the electron wavefunctions in the CQD films.

In addition, it has been reported that ligand exchange using halide ions does exhibit high *J*_sc_ compared to other short molecular length organic ligands, but that a strong compromise exists in the form of the formation of sub-ε_gap_ states. It is also noted that these sub-ε_gap_ states are mainly generated from the weak dangling bonds between the original ligands or partially charged Pb atoms after the ligand exchange process.^21^ In the present work, after employing the HB treatment, pyridine atoms will form an extra level of bonding with the partially charged Pb atoms, which will suppress the occurrence of the sub-ε_gap_ states. Compared with TBAI-only films, the increase in the *V*_oc_ after the HB passivation treatment also supports our claims. To further investigate the oxidation states of the PbS CQDs (Fig. S3†), X-ray photoelectron spectroscopy (XPS) was performed to probe the Pb charge states on the surface of the CQDs for both the HB and TBAI treated films. We observed that the Pb 4f peaks with the HB passivation have a higher binding energy and smaller FWHM values than those observed for the TBAI passivation films. Therefore, the XPS peak positions for HB passivation are much closer to the expected Pb–S bond feature whereas films with only the TBAI passivation appear to be much closer to that of metallic Pb. This suggests that the HB treatment process plays a key role in maintaining the Pb–S bond state, which prevents the generation of the dangling bonds and charged Pb atoms enabling the suppression of the formation of the CQD sub-ε_gap_ states. Moreover, its smaller FWHM value indicates that multiple oxidation states are less prevalent than for the TBAI treatment.

## Conclusion

We have demonstrated that the performance of CQD solar cells can be effectively manipulated by using organic/inorganic hybrid passivation strategies. The resulting high performance CQD solar cell devices have been successfully fabricated by employing a hybrid organic–inorganic method. The shortest amine base (pyridine) can result in a near complete removal of oleic acids and reduce surface trap sites on CQD films. Moreover, spectroscopy and photoresponse measurement indicate that the CQD films with a hybrid (HB) passivation exhibit closer CQD spacing, uniform film fabrication, reduced PL quenching and longer PL decay, and an enhanced photoresponse. We have demonstrated that the extra binding of the pyridine molecule on the CQD surface can reduce the recombination losses and improve the charge transfer in quantum dot films. In doing so, the use of hybrid CQD films in solar cell devices resulted in improved *V*_oc_ and *J*_sc_ at the expense of the FF. Conclusively, we have increased the power conversion efficiency of the solar cell from 5.3% to 6.8% using the hybrid passivation treatments. The HB surface treatment on CQD films is a promising technique for producing highly efficient CQD devices for a range of optoelectronic applications such as solar cells. Moreover, this approach can be used to help better understand the energy harvesting dynamics and the charge carrier transfer dynamics in CQDs.

## Author contribution

J. H, B. H. and S. C. carried out experimental design, experiments and data analyses. J. L., S. P., B. K., Y. L., S. P. and J. P. carried out structural and photovoltaic analyses. P. G., S. M., H. S., J. S. and J. K. contributed to the scientific discussion and planned the work and provided experimental guidance. J. H., B. H. and S. C. wrote the manuscript and all authors reviewed the manuscript.
