# Supplementary material for: Enhanced charge carrier transport properties in colloidal quantum dot solar cells via organic and inorganic hybrid surface passivation
Source: J Mater Chem A Mater. 2016 Oct 7;4(48):18769–75. doi: 10.1039/c6ta06835a (PMC5735354; doi:10.1039/c6ta06835a)
Supplement: Supplementary file 1 [file TA-004-C6TA06835A-s001.pdf]

Electronic Supplementary Material (ESI) for Journal of Materials Chemistry A.
This journal is © The Royal Society of Chemistry 2016

Supporting Information

Enhanced charge carrier transport properties in colloidal quantum dot solar cells via organic and inorganic hybrid surface passivation

John Hong[1§], Bo Hou[1§], Jongchul Lim[2], Sangyeon Pak[1], Byung-Sung Kim[1], Yuljae Cho[1], Juwon Lee[1], Young-Woo Lee[1], Paul Giraud[1], Sanghyo Lee[1], Jong Bae Park[3], Stephen M. Morris[1], Henry J. Snaith[2], Jung Inn Sohn[1]*, SeungNam Cha[1]*, Jong Min Kim[4]

[1] *Department of Engineering Science, University of Oxford, Oxford OX1 3PJ, United Kingdom.*

[2] *Department of Physics, Clarendon Laboratory, University of Oxford, Oxford OX1 3PU, United Kingdom.*

[3] *Jeonju Centre, Korea Basic Science Institute, Jeonju, Jeollabuk-do 561-180, Republic of Korea.*

[4] *Department of Engineering, University of Cambridge, Cambridge CB3 0FA, United Kingdom.*

* *Corresponding authors (junginn.sohn@eng.ox.ac.uk, seungnam.cha@eng.ox.ac.uk)*

§ *These authors contributed equally to this work*

Experimental Section

PbS Colloidal QD (1.28eV) Synthesis.

PbS CQDs with an absorption peak at 980nm(1.28eV) were synthesized using the method described elsewhere.[1] A lead precursor solution was produced by mixing lead oxide (PbO), oleic acids (OAs) and 1-octadecene (ODE) in a 50 ml two neck flask and then heated to 100 °C under vacuum for 3hr. For further treatment, the lead precursor solution was then heated to 130 °C for 1hr under argon. In addition, a sulfur precursor solution was produced by mixing hexamethyldisilathiane (TMS) and ODE in a 50 ml two neck flask and treated at room temperature for 4hr under argon. The PbS CQD synthesis was completed by rapid injection of the sulfur precursor solution in to the flask of the lead precursor solution. The PbS CQDs were purified by a few centrifuge steps. Further wash steps were applied to the extracted PbS CQDs and the PbS CQDs were finally dispersed in a toluene solvent to produce a 50 mg ml^{-1} concentration.

ZnO Nanoparticle Solution Synthesis.

ZnO nanoparticles were synthesized using the method described elsewhere.[2] A Zn precursor solution was produced by dissolving 0.9788 g of zinc acetate dihydrate in methanol that was heated to 60 °C in a flask. Potassium hydroxide (KOH) was then dissolved in 22 ml of methanol and dropped into the flask for 20 min. The ZnO solution was kept at 60 °C until it turned into a clear solution. The ZnO solution was then cooled to room temperature and purified. Finally, the ZnO NPs were dispersed in a chloroform solvent to produce a 50 mg ml^{-1} concentration.

Photovoltaic Device Fabrication.

Patterned indium tin oxide (ITO) substrates were cleaned with acetone, ethanol and 1-propanol solvents and finally treated with oxygen plasma for 4 min to remove any residue on the

substrates. After cleaning, the ZnO nanoparticle solution was spin coated for 30 sec and annealed at 100 °C for 30 min so as to deposit a highly crystallized ZnO electron transfer film. For the quantum dot photovoltaic layers with the TBAI passivation, a drop of the PbS CQD solution was deposited onto spinning substrates with a spin rate of 2500 rpm. 5 drops of the TBAI solution (10 mg ml^{-1} in methanol) was then dropped onto the CQD films and then left for 30 secs before 4 drops of methanol were applied to remove the excess TBAI solution on the CQD films. For the HB (TBAI + pyridine) CQD films, the same fabrication method was used, but during the methanol cleaning steps, a few drops of the pyridine solution (0.5 vol% in acetonitrile) were also applied to the CQD films. After the CQD film fabrication, Au top electrodes were deposited on the substrates by thermal evaporation with 100 nm thickness. For the photodetector devices, an Au top electrode was deposited on to 300nm SiO_2/p-type Si wafers by thermal evaporation with 100 nm thickness under a patterned mask, and quantum dot films (3 layers) of HB and TBAI were spin coated on to the Au/SiO_2/p-type Si wafers.

Device Characterization.

CQD photo-devices were fabricated using a PbS solution and ligand exchange process by standard photolithography. Photoresist (MICROPOSIT S1813) was spin coated on a silicon wafer with 300 nm SiO_2 layer and baked at 110 °C for 60 sec. The substrate was exposed through a photomask of the electrode pattern and then developed in MICROPOSIT MF-319 developer for 30 sec and finally the silicon substrate was then rinsed with water for 2 min. A metal electrode of 100 nm Au was deposited by thermal evaporation. PbS solutions and the same ligand exchange process for TBAI and HB passivation were deposited onto the substrate using a layer-by-layer spin coating method. 5 layers of CQD films were deposited. Photoresponse was detected by focusing the light onto the quantum photodetector devices with a 635 nm light source (LDM635/1LJM) with a power of 1 mW . The time period of the laser

source was 500 ms, and the on and off delay time was 1.8 μs. The photoresponse measurements were carried out using a probe station (Cascade Microtech) and Keithley 4200-SCS system.

Characterization of semiconductor CQDs.

Transmission electron microscopy (TEM) measurement was carried out by TEM spectroscopy (JEM-2200MCO FEGTEM, JEOL). Morphology investigations of the PbS films were measured by atomic force microscopy (Veeco Dimension 3100). Fourier transform infrared spectroscopy (FT-IR) was carried out using a Varian Excalibur FTS 3500. FT-IR samples of the HB and TBAI CQD films were prepared on the glass windows, and the FT-IR measurements were operated under room temperature conditions. Steady-state photoluminescence(PL) measurements were carried out by an automated spectrofluorometer (Fluorolog, Horiba Jobin Yvon) with a 450 W xenon lamp excitation source and an NIR detector. Time-resolved photoluminescence(TR-PL) measurements were carried out using a time-correlated single photon counting apparatus (FluoTime 300, PicoQuant GmbH) with a NIR-PMT detector assembly (H10330A-45, Hamamatsu).

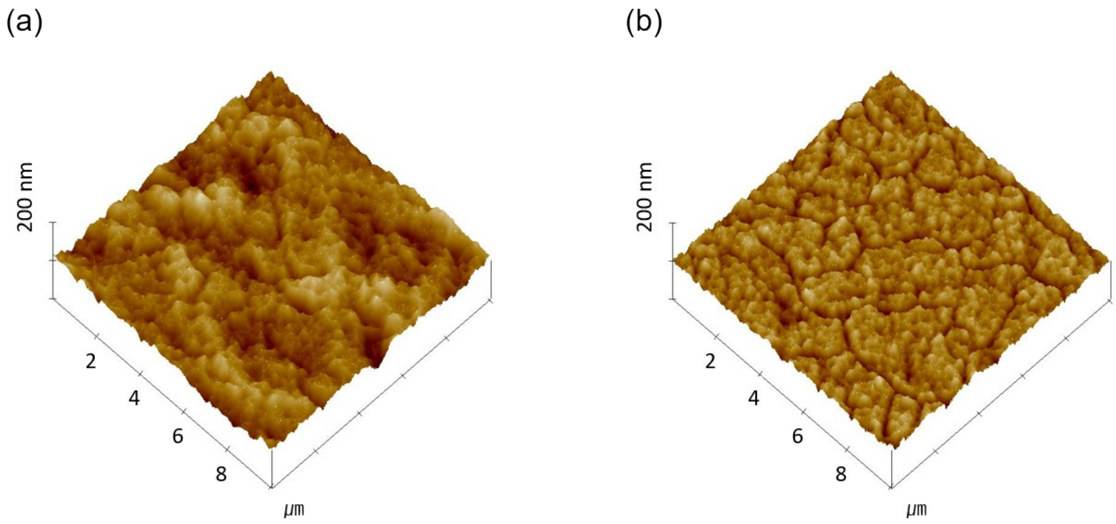

Figure S1. AFM 3D imaging of CQD films with (a) TBAI and (b) HB passivation.

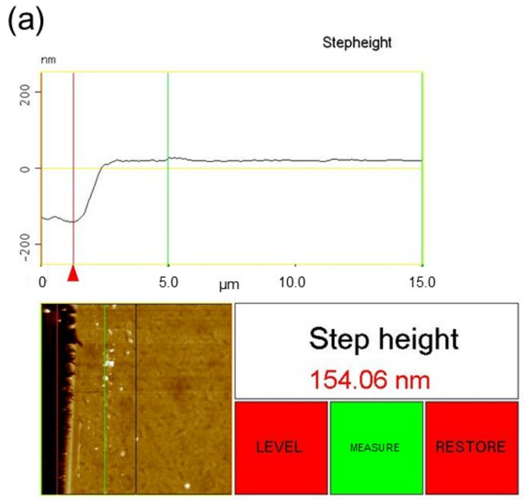

Figure S2. Film thickness of CQD films with (a) TBAI and (b) HB passivation by AFM.

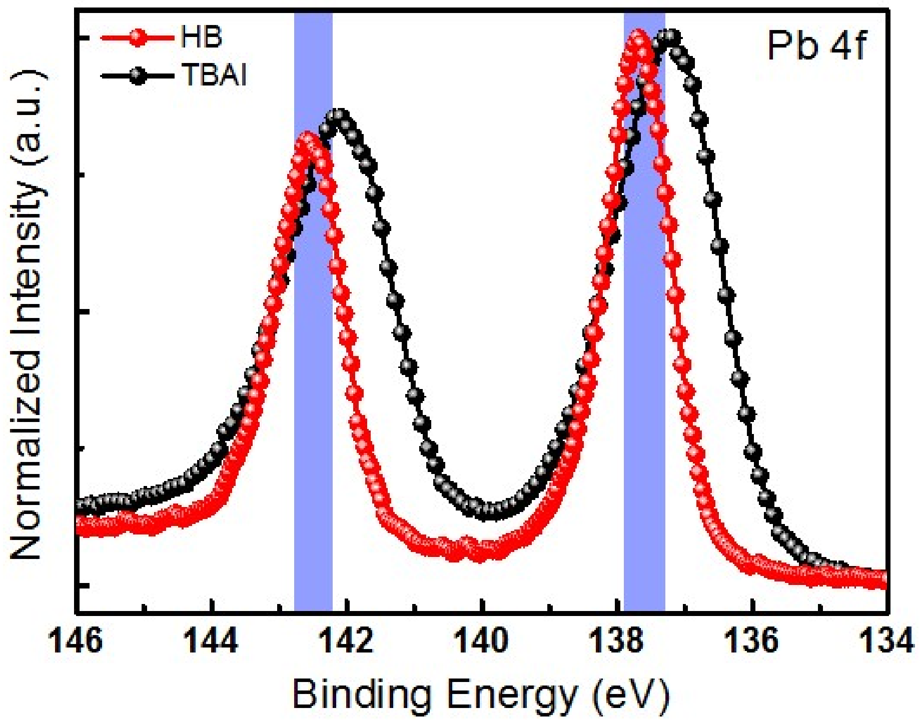

Figure S3. XPS spectra of Pb 4f in the CQD films with TBAI and HB passivation.

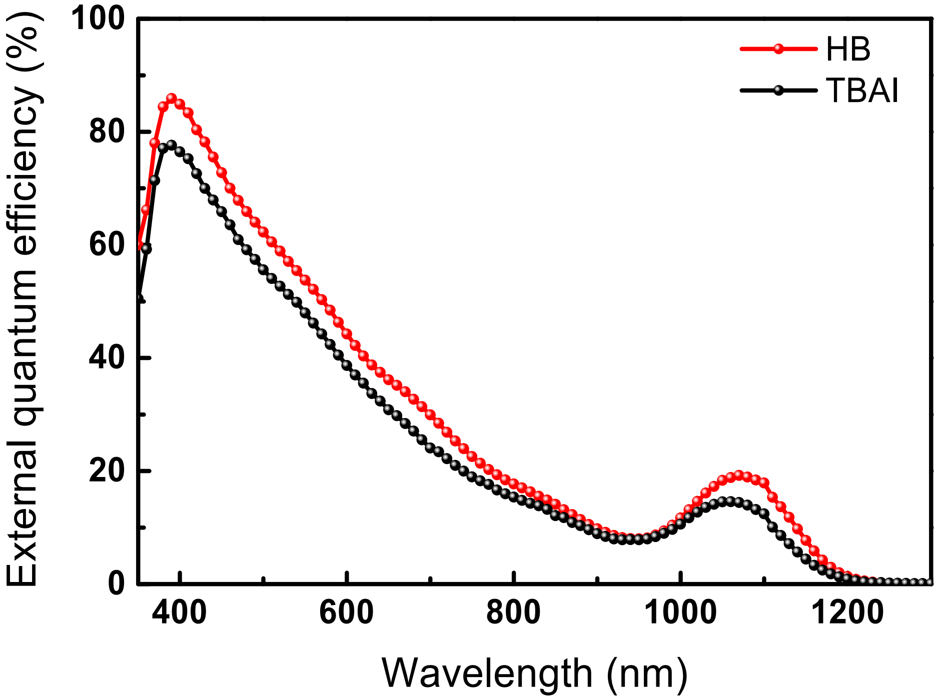

Figure S4. External quantum efficiency(EQE) as a function of wavelength for the HB and TBAI films

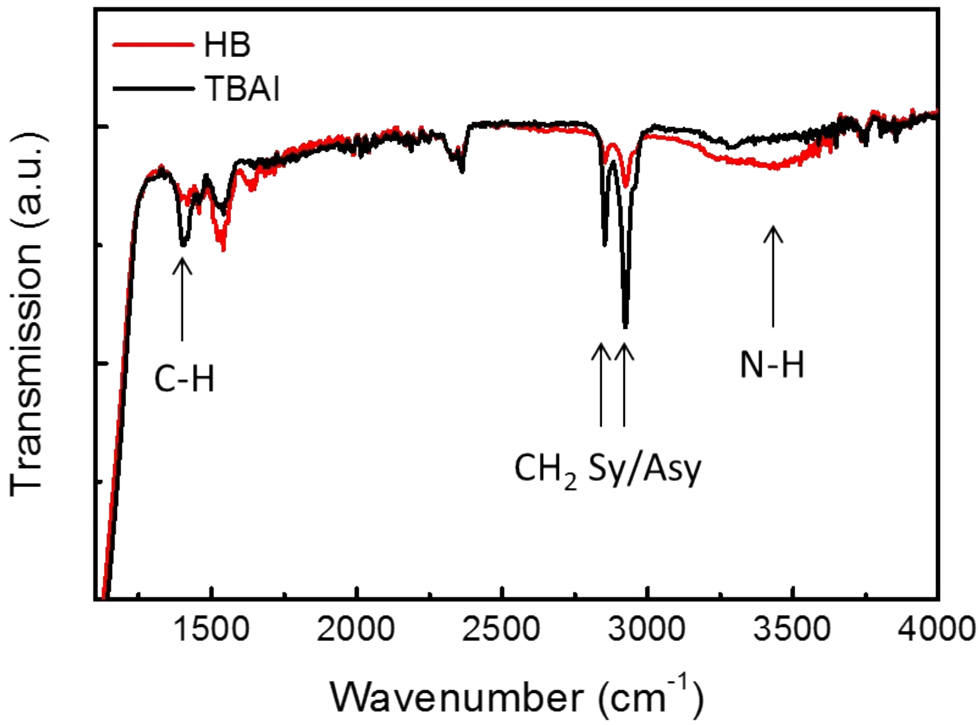

Figure S5. Full spectral range of the HB and TBAI CQD films for a 1300~4000 nm range.

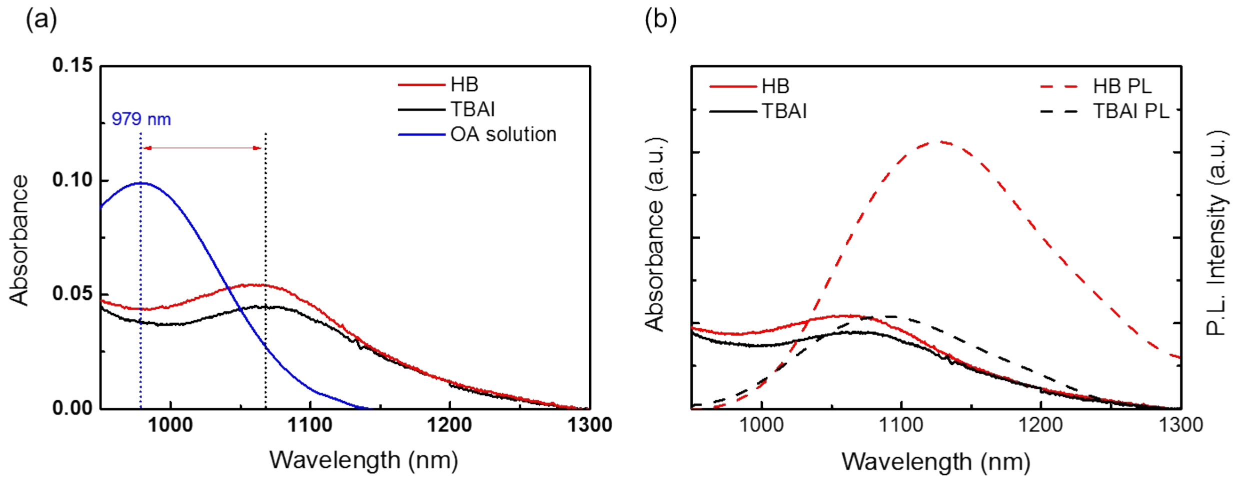

Fig. S6. (a) UV-vis absorption spectra of PbS OA solution (black), HB(Red) and TBAI (blue) CQD films. The FWHM of the first exciton peak for the HB and TBAI CQD films are 160.17nm and 178.71nm, respectively. (b) Comparison between absorption edge and luminescence spectra of the HB and TBAI CQD films

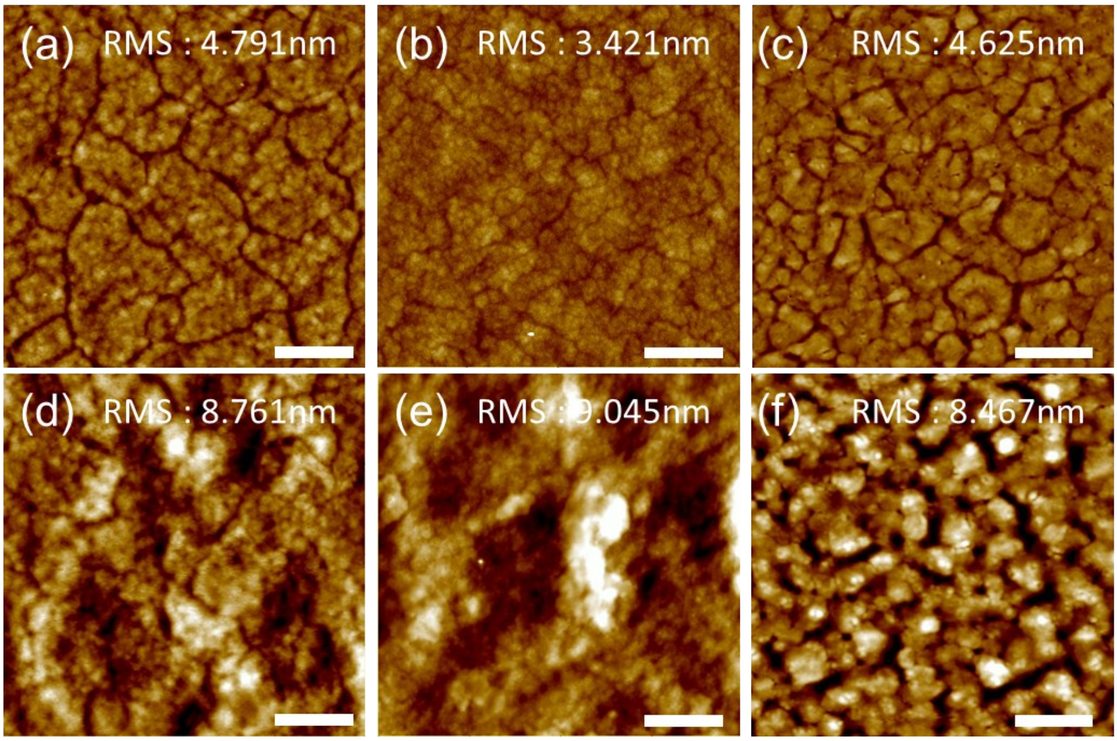

Figure S7. AFM images of the (a-c) HB and (d-f) TBAI-treated CQD films. Scale bars are 2μm.

RMS values with different locations	HB films (nm)	TBAI films (nm)
1	4.791	10.195
2	3.421	8.163
3	4.888	8.467
4	4.625	8.278
5	4.497	9.045
6	5.087	11.989
7	3.721	8.163
8	4.691	8.761
Average	4.465	9.132

Table S1. Average AFM RMS values of the HB and TBAI films. 8 different locations are selected from the active solar cell films.

References

[1] M. A. Hines and G. D. Scholes, *Adv. Mater.*, 2003, **15**, 1844–1849.

[2] W. J. Beek, M. M. Wienk, M. Kemerink, X. Yang, and R. A. A. Janssen, *J. Phys. Chem. B*, 2005, **109**, 9505–9516.
